# Decreased S100A9 Expression Promoted Rat Airway Smooth Muscle Cell Proliferation by Stimulating ROS Generation and Inhibiting p38 MAPK

**DOI:** 10.1155/2016/1462563

**Published:** 2016-12-05

**Authors:** Lei-Miao Yin, Xiao-Jie Han, Ting-Ting Duan, Yu-Dong Xu, Yu Wang, Luis Ulloa, Yong-Qing Yang

**Affiliations:** ^1^Laboratory of Molecular Biology, Shanghai Research Institute of Acupuncture and Meridian, Yue Yang Hospital, Shanghai University of Traditional Chinese Medicine, Shanghai 200030, China; ^2^Center of Immunology and Inflammation, Rutgers-New Jersey Medical School, Rutgers University, Newark, NJ 07101, USA

## Abstract

*Background*. Asthma is a disease with a core abnormality in airway smooth muscle function, and the proliferation of airway smooth muscle cells (ASMCs) plays a pivotal role in asthma airway remodeling. Our previous study showed that S100A9 (S100 calcium-binding protein A9; 400 and 800 ng/mL) significantly inhibited rat ASMCs proliferation at 48 h, and 50–800 ng/mL S100A9 (50, 100, 200, 400, and 800 ng/mL) also induced a lasting effect by significantly inhibiting rat ASMCs proliferation at 72 h in a dose-dependent manner. However, the intracellular effects of S100A9 on ASMCs proliferation remain unknown.* Methods*. Rat ASMCs with stable S100A9 knockdown were generated using short hairpin RNA. The effects of decreased S100A9 expression on cellular proliferation, the production of reactive oxygen species (ROS), and p38 MAPK pathway protein expression were examined.* Results*. Decreased intracellular S100A9 expression significantly promoted platelet-derived growth factor-induced rat ASMCs proliferation and increased ROS production. The antioxidative agent N-acetylcysteine significantly inhibited rat ASMCs proliferation. Western blot results showed that the decreased intracellular S100A9 expression significantly inhibited p38 MAPK phosphorylation.* Conclusion*. Decreased S100A9 expression promoted rat ASMCs proliferation by stimulating ROS generation and inhibiting p38 MAPK. Our study may provide novel insights into the regulation of asthma airway remodeling.

## 1. Introduction

Asthma is a noninfectious chronic disease that affects as many as 334 million people worldwide [1]. The World Health Organization (WHO) has estimated that 15 million disability-adjusted life-years are lost annually and that 250,000 asthma deaths occur globally [2]. Asthma is now recognized as a disease of major public health importance due to its increasing prevalence, morbidity, and mortality in recent decades [3].

Asthma is a disease with a core abnormality in airway smooth muscle cells (ASMCs) [4]. ASMCs proliferation plays a key role in asthma airway remodeling and involves various signal transduction pathways and mediators, such as reactive oxygen species (ROS) and p38 mitogen-activated protein kinase (MAPK) [5, 6]. ROS can act as second messengers to regulate different signaling pathways, and they are important mediators of cellular proliferation [7]. Excessive mitochondrial ROS levels lead to enhanced inflammatory responses in the lung and ASMCs proliferation [8]. Cigarette smoke extract enhances human ASMCs proliferation by increasing ROS generation and cytokine secretion [9]. TGF-*β*1 stimulates Nox4 expression and increases ROS production to facilitate the proliferation of human airway smooth muscle cells [10]. Furthermore, platelet-derived growth factor- (PDGF-) induced ASMCs proliferation is closely related to ROS production [11]. On the contrary, the antioxidant catalase significantly reduces PDGF-stimulated ASMCs proliferation [12], and antioxidants such as catalase, N-acetylcysteine, and superoxide dismutase significantly decrease the proliferation of human ASMCs [13]. As important mediators of signal transduction, p38 MAPK integrates signals that affect proliferation and survival in a cell context- and type-specific manner [14]. p38 MAPK generally exerts antagonistic effects on cell proliferation and survival [14]. It was reported that p38 MAPK participates in the basic fibroblast growth factor- (bFGF-) stimulated ASMCs proliferation as this effect is prevented by the kinase inhibitor SB 203580 [15]. p38 MAPK signaling pathway is significantly inhibited by PDGF-induced proliferation in smooth muscle cells. For example, astragaloside IV inhibits PDGF-stimulated proliferation by inhibiting p38 MAPK in human vascular smooth muscle cells [16]. p38 MAPK activation is also required for the esculetin-induced inhibition of vascular smooth muscle cell proliferation [17].

ASMCs proliferation could also be regulated by S100A9 (S100 calcium-binding protein A9) which plays an important role in asthma pathogenesis and morbidity [18]. Proteomics study showed that S100A9 in sputum could be a potential biomarker of neutrophilic inflammation in severe uncontrolled asthma [19]. S100A9 has also been identified as a key mediator in mucin hyperproduction of neutrophil-dominant airway inflammation [20]. In asthmatic patients, S100A9 expression was downregulated in sputum [21]. However, S100A9 level was elevated in serum of bakery workers and involved in innate immune responses of baker's asthma pathogenesis [22]. Our previous study showed that S100A9 is one of the differentially expressed genes in a rat lung model of asthma [23]. It elicits dose-dependent antiasthmatic effects [24]. S100A9 (400, 800 ng/mL) significantly inhibited rat ASMCs proliferation at 48 h, and S100A9 (50, 100, 200, 400, and 800 ng/mL) significantly inhibited rat ASMCs proliferation at 72 h [25]. S100A9 expression level appears to play a role in asthma, but a definitive study on the intracellular regulatory effects of S100A9 on ASMCs proliferation is lacking.

Due to the expression of S100A9 in ASMCs, the S100A9 level was silenced using short hairpin RNA (shRNA). Our study aimed to determine the effect of decreased S100A9 levels on ASMCs proliferation, which could potentially provide new insight into the regulation of asthma airway remodeling.

## 2. Materials and Methods

### 2.1. Preparation of Primary Cultured Rat ASMCs

ASMCs were isolated and cultured according to our previous description [26]. Briefly, a rat trachea was placed into sterile, ice-cold, HPPS solution (130.0 mM NaCl, 5.0 mM KCl, 1.2 mM MgCl_2_·6H_2_O, 10.0 mM HEPES, and 10.0 mM glucose, pH = 7.4), and the surrounding undesired tissue was dissected. The trachea was cut into small segments and digested for 30 min at 37°C in HPPS solution containing 2.0 mg/mL collagenase IV. Enzyme digests were subsequently centrifuged at 1000 rpm for 5 min, and the pellet was resuspended and cultured in DMEM supplemented with 10% FBS. The ASMCs were confirmed by light microscopy and immunofluorescence confirmed that more than 95% of the cells in the primary culture expressed the contractile protein SM *α*-actin. The experiments were performed with cells at passages 3–9.

### 2.2. Stable Knockdown of S100A9 in Rat ASMCs

Using the BLOCK-iT RNAi system (Invitrogen, USA), double-stranded oligonucleotides were designed to form an engineered pre-miRNA structure that targets S100A9 (S100A9 RNAi; see Table 1). The synthesized oligonucleotides were annealed and ligated into pcDNA 6.2-GW/EmGFP-miR. The EmGFP-miRNA cassette in this construct was subsequently subcloned using the pAd-CMV-Dest Gateway Vector Kit. Virus was produced in a packaging cell line (293A) using an expression system (ViraPower Adenoviral Expression System; Invitrogen) according to the manufacturer's protocol. Primary cultured rat ASMCs were transfected with vector (Pac I-digested pAd-CMV-Dest) containing the desired RNAi cassette using Lipofectamine 2000. Mature viral particles were harvested by collecting the cells, subjecting them to multiple freeze-thaw cycles, and then centrifuging them. GFP expression indicated S100A9 RNAi expression. Efficient knockdown was attained 72 h after virus addition. Stable knockdown of S100A9 expression in rat ASMCs (ASMC-shRNA-S100A9) was confirmed by qRT-PCR and Western blot.

### 2.3. qRT-PCR Analysis

S100A9 gene expression was analyzed by qRT-PCR using the LightCycler 96 Real-Time PCR System (Roche Applied Science) and TOYOBO Real-Time PCR Master Mix. The forward primer for S100A9 was 5′-ACCCTGAACAAGGCGGAATT-3′, and the reverse primer was 5′-TTTGTGTCCAGGTCCTCCATG-3′. The expression ratio was calculated according to the 2^−ΔΔCt^ method [27]. Transcripts with a 2-fold increase in expression were considered to be upregulated, and those with a 0.5-fold decrease in expression were considered to be downregulated.

### 2.4. WST-1 Assay

ASMC-shRNA-S100A9 cells were harvested by trypsinization and resuspended in DMEM containing 10% FBS. The cells were then plated at a density of 5,000 cells per well in 96-well plates and incubated overnight. Next, the cells were stimulated with 25 ng/mL PDGF in 1% FBS-DMEM for 24 h, 48 h, or 72 h. The WST-1 assay was conducted as previously described [24]. Briefly, 10 *μ*L of WST-1 reagent was added to each well containing 90 *μ*L of cell suspension at each time point, and the mixture was incubated for an additional 2 h. The absorbance at 450 nm was monitored with a reference wavelength of 630 nm. Cell proliferation in each group was calculated by comparison with the control group.

### 2.5. Label-Free, Impedance-Based Real-Time Cellular Assay

Proliferation was also measured using an xCELLigence Real-Time Cell Analyzer (RTCA, ACEA Biosciences, USA), which measures electrical impedance as a readout for the barrier status of cells grown directly on biocompatible microelectrode-coated surfaces. Changes in impedance (represented as the cell index) reflect changes in proliferation, and the assays were conducted as previously described [28]. Briefly, the cells were seeded into E-Plate 16 devices at a density of 3000 cells/well with DMEM containing 10% FBS. Then, the cells were stimulated with 25 ng/mL PDGF in 1% FBS-DMEM for 24 h, 48 h, or 72 h. The effect of an antioxidative agent N-acetylcysteine (NAC) on ASMCs proliferation was investigated by RTCA system. 10 mM NAC was added in 1% FBS-DMEM for 24 h, 48 h, or 72 h. The impedance readings were taken automatically every 15 min and data were generated from ASMCs with 4 replicates.

### 2.6. Oxidation Assay

This assay is based on the ROS-dependent oxidation of 2′-7′-dichlorodihydrofluorescein diacetate (DCFH-DA) to 2′-7′-dichlorofluorescein (DCF). Briefly, serum-free medium (SFM) containing DCFH-DA (final concentration 10 *μ*g/mL) was added to the cell culture dish (ASMCs at 80% confluence), and the cells were incubated at 37°C for 25 min. After removing the SFM and washing the cells 3 times with PBS, 1 mL of PBS was added to the culture dish and the cellular fluorescence was visualized using a fluorescent microscope (Olympus IX-81, Japan) with an excitation of 485 nm, an emission of 530 nm, and an exposure time of 400 ms. The fluorescence of forty cells from three microscope fields was captured, digitized, and measured using Xcellence RT microscope imaging software. The assay was repeated in triplicate and reported as the ratio of DCFH-DA fluorescence to cell area.

### 2.7. Western Blot Analysis

To investigate the protein changes related to decreased S100A9 expression in ASMCs, the cells were lysed (50 mM HEPES, 150 mM NaCl, 1 mM EDTA, 10% glycerol, 1% Triton X-100, 25 mM NaF, 10 *μ*M ZnCl_2_, and protease and phosphatase inhibitor tablets (Roche), pH = 7.5) and incubated on ice for 60 min and centrifuged at 10,000 ×g for 10 min to obtain the cell-free extracts. Equal amounts of protein were separated by SDS-PAGE, transferred to PVDF membranes, and analyzed using the following specific primary antibodies: S100A9 (ab75478; Abcam), p-p38 MAPK (Thr180/Tyr182, 4511; CST, USA), p38 MAPK (8690; CST), and *β*-actin (4967; CST). Horseradish peroxidase- (HRP-) conjugated secondary antibodies were applied, and the membrane was then developed using the ECL detection system.

### 2.8. Statistical Analysis

All data are presented as the mean ± SD. Significant differences between two data sets were assessed with two-tailed Student's *t*-test. Statistical significance between different groups in WB analysis was calculated using one-way ANOVA followed by LSD post hoc test. *P* values less than 0.05 were considered to indicate statistical significance.

## 3. Results

### 3.1. Effect of Decreased S100A9 Expression on ASMCs Proliferation

**Figure 1:**
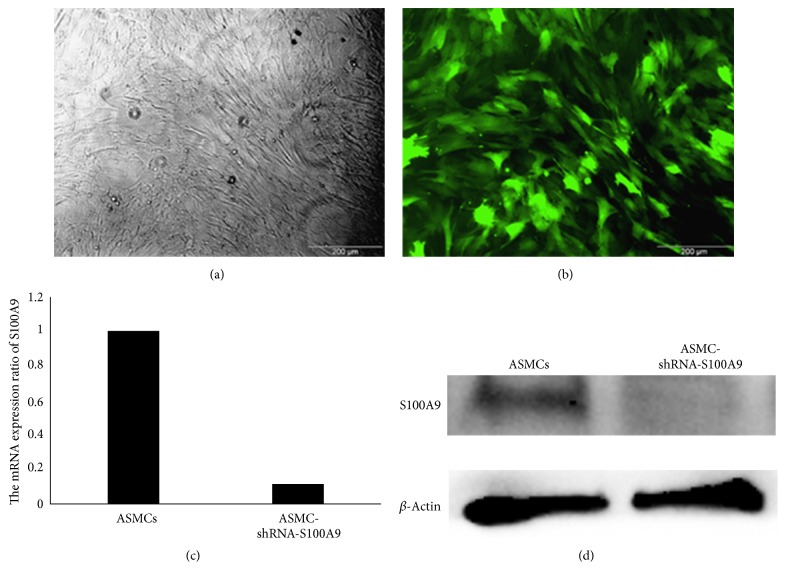
Generation of airway smooth muscle cells (ASMCs) with stable knockdown of S100A9 expression (ASMC-shRNA-S100A9). (a) Light microscopy image of ASMC-shRNA-S100A9 cells. (b) Green fluorescent protein (GFP) expression indicated the expression of S100A9 RNAi, and GFP-positive cells accounted for more than 95% of the total cell population by fluorescence microscopy. (c) Ratio of S100A9 mRNA expression in ASMC-shRNA-S100A9 cells compared with control ASMCs. (d) Western blots showed that S100A9 protein expression was significantly decreased in ASMC-shRNA-S100A9 cells.

GFP protein expression confirmed that S100A9 RNAi was expressed and GFP-positive cells accounted for more than 95% of the total cell population (Figures 1(a) and 1(b)). qRT-PCR showed that S100A9 mRNA levels decreased to 0.11 in ASMCs exposed to the 12MR0174-2 sequence compared with control ASMCs (Figure 1(c)). Western blots showed that S100A9 protein expression was significantly decreased in ASMC-shRNA-S100A9 cells (Figure 1(d)).

**Figure 2:**
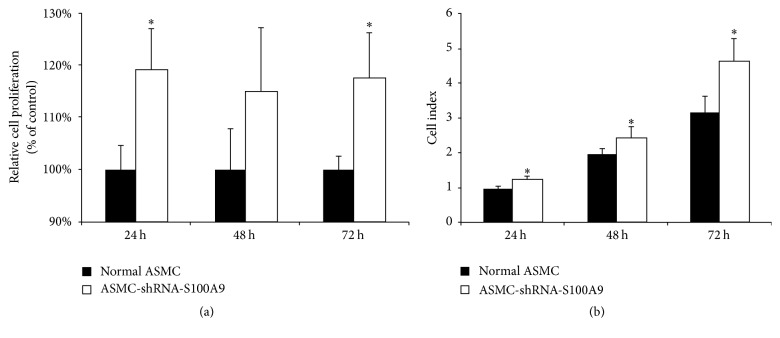
Effect of stable knockdown of S100A9 expression on airway smooth muscle cellular proliferation. (a) PDGF-induced proliferation in ASMC-shRNA-S100A9 cells measured by the WST-1 assay at 24 h, 48 h, and 72 h. (b) PDGF-induced proliferation in ASMC-shRNA-S100A9 cells measured by the real-time cellular assay at 24 h, 48 h, and 72 h. *∗* indicates a significant difference as compared to control ASMCs (*P* < 0.05). All data are presented as the means ± SD, *n* = 4.

The effect of decreased S100A9 expression on the proliferation of ASMCs and ASMC-shRNA-S100A9 cells was assessed by both WST-1 and RTCA assays. Without stimulation, ASMCs and ASMC-shRNA-S100A9 cells show no significant difference in proliferation. However, the WST-1 assay indicated that the PDGF-induced proliferation of ASMC-shRNA-S100A9 was significantly increased at 24 and 72 h (Figure 2(a), *P* < 0.05). The RTCA assay confirmed that the PDGF-induced proliferation of ASMC-shRNA-S100A9 cells was significantly increased at 24, 48, and 72 h compared with control cells (Figure 2(b), *P* < 0.05).

### 3.2. Effect of Decreased S100A9 Expression on ROS Production

**Figure 3:**
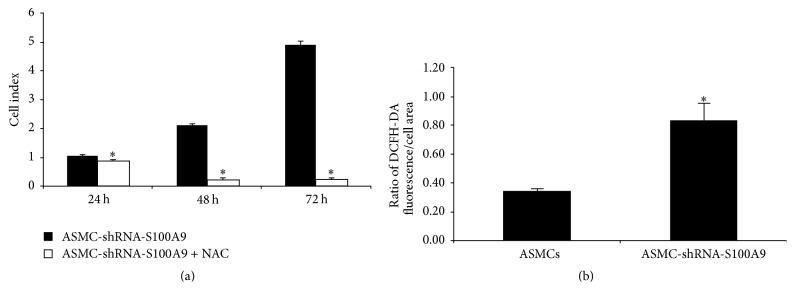
Quantification of oxidative stress in airway smooth muscle cells (ASMCs) with stable knockdown of S100A9 expression (ASMC-shRNA-S100A9). (a) Effects of antioxidative agent N-acetylcysteine (NAC) on ASMC-shRNA-S100A9 proliferation at 24 h, 48 h, and 72 h. *∗* indicates a significant difference as compared to control (*P* < 0.05). (b) Comparison of the ratio of DCFH-DA fluorescence to cell area in ASMCs and ASMC-shRNA-S100A9 cells. The fluorescence of forty cells from three microscope fields was measured and the assay was repeated in triplicate. *∗* indicates a significant difference as compared to ASMCs (*P* < 0.05). All data are presented as the means ± SD.

Then, we examined the effect of ROS on the proliferation of ASMC-shRNA-S100A9. Antioxidant NAC significantly decreased ASMC-shRNA-S100A9 proliferation (Figure 3(a), *P* < 0.05). The effect of intracellular S100A9 expression on ROS production was also determined. The ratio of DCFH-DA fluorescence to cell area was 0.35 ± 0.02 for control ASMCs and 0.84 ± 0.12 for ASMC-shRNA-S100A9 cells, indicating a significant increase in intracellular ROS (Figure 3(b), *P* < 0.05, *n* = 3).

### 3.3. Effect of Decreased S100A9 Expression on p38 MAPK

**Figure 4:**
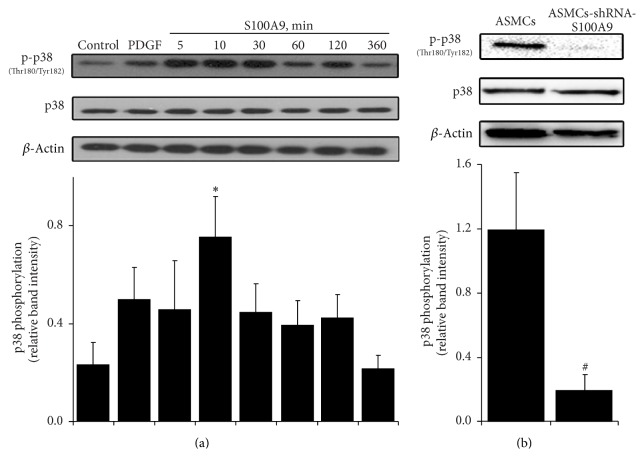
Western blot analysis of the effects of extracellular and intracellular S100A9 on p38 MAPK. (a) Effect of the addition of S100A9 on p38 MAPK in control airway smooth muscle cells (ASMCs). *∗* indicates a significant difference as compared to control (*P* < 0.05). (b) Effect of stable knockdown of S100A9 expression in ASMCs (ASMC-shRNA-S100A9) on p38 MAPK. # indicates a significant difference as compared to ASMCs.

The effect of extracellular S100A9 on p38 MAPK was also examined using Western blot analysis. Extracellular S100A9 protein (800 ng/mL) significantly induced p38 phosphorylation in ASMCs. The results showed that p38 phosphorylation was significantly increased after a 10 min incubation with extracellular S100A9 (Figure 4(a)). Furthermore, reduced intracellular S100A9 expression significantly decreased p38 MAPK phosphorylation in ASMC-shRNA-S100A9 cells (Figure 4(b)). These data suggested that S100A9 affected p38 phosphorylation that may be related to the proliferation of ASMCs.

## 4. Discussion

Our study showed that the decreased intracellular S100A9 expression significantly promoted ASMCs proliferation, suggesting that S100A9 expression levels are closely related to ASMCs proliferation, which is consistent with our extracellular examination [25]. Although there are conflicting reports concerning S100A9 function as a pro- or anti-inflammatory factor, the actual biological functions of S100A9 may depend on several factors, including cell type, pro- and anti-inflammatory mediators, and receptors [29].

ROS are essential mediators of cell proliferation and our result confirmed that antioxidative agent NAC significantly inhibited ASMCs proliferation. The role of S100A9 in the ROS regulation was subsequently investigated. The results showed that the decreased intracellular S100A9 expression significantly promoted ROS production which are directly related to ASMCs proliferation. Consistently, S100A9 has been considered one of the most important oxidant scavengers in inflammation and the biological functions of S100A9 may likely be attained through ROS regulation [30]. It was reported that S100A9 inhibits polymorphonuclear neutrophil oxidative metabolism and significantly reduced ROS production [31]. S100A9 has also been shown to scavenge ROS to potentially reduce oxidative stress [32]. Our results suggest that decreased intracellular S100A9 expression enhanced PDGF-induced ASMCs proliferation by promoting ROS production.

The effect on the signaling pathways of extracellular S100A9 revealed an increased phosphorylation of p38 MAPK [33]. Our previous results also confirmed that 800 ng/mL S100A9 significantly induced p38 MAPK phosphorylation. Therefore, p38 MAPK phosphorylation was further evaluated in this study. The results showed that decreased S100A9 expression significantly inhibited p38 MAPK phosphorylation in ASMC-shRNA-S100A9 cells, suggesting that the antiproliferative effects of S100A9 in ASMCs may be related to p38 MAPK phosphorylation.

## 5. Conclusions

In summary, decreased S100A9 expression promoted rat ASMCs proliferation by stimulating ROS generation and inhibiting p38 MAPK, which may provide new insights into the regulation of asthma airway remodeling.

## Figures and Tables

**Table 1:** Oligo-DNA sequences for rat S100A9.

Name	Oligo-DNA sequence (5′-3′)
12MR0174-1	TGCTGTTACTTCCCACAGCCTTTGCCGTTTTGGCCACTG
Forward	ACTGACGGCAAAGGGTGGGAAGTAA
12MR0174-1	CCTGTTACTTCCCACCCTTTGCCGTCAGTCAGTGGCCAA
Reverse	AACGGCAAAGGCTGTGGGAAGTAAC
12MR0174-2	TGCTGAAATTTGGCAAGTCCTTATTCGTTTTGGCCACTG
Forward	ACTGACGAATAAGGTTGCCAAATTT
12MR0174-2	CCTGAAATTTGGCAACCTTATTCGTCAGTCAGTGGCCAA
Reverse	AACGAATAAGGACTTGCCAAATTTC
12MR0174-3	TGCTGTGATGATGGTGCTTATGCTGCGTTTTGGCCACTG
Forward	ACTGACGCAGCATACACCATCATCA
12MR0174-3	CCTGTGATGATGGTGTATGCTGCGTCAGTCAGTGGCCA
Reverse	AAACGCAGCATAAGCACCATCATCAC
12MR0174-4	TGCTGCTCAAAGGACAGTTGATTGTCGTTTTGGCCACTG
Forward	ACTGACGACAATCATGTCCTTTGAG
12MR0174-4	CCTGCTCAAAGGACATGATTGTCGTCAGTCAGTGGCCA
Reverse	AAACGACAATCAACTGTCCTTTGAGC
